# Editorial

**DOI:** 10.1120/jacmp.v6i3.2172

**Published:** 2005-08-17

## Transaction Costs and Scholarly Publishing

The Journal of Applied Clinical Medical Physics (JACMP) is here because of a revolution in publishing technology. That publishing revolution has greatly lowered some of the costs of publishing scholarly articles. However, this is not true of all costs across the board, as some do not change. For example, it costs as much for copy‐editing, layout production and quality assurance services for the JACMP as for any print journal. Still, take a look as some of the costs associated with a print and electronic journal:

- A library will typically pay $1.00 per print article per year to obtain, own and archive that article
- A library will typically pay far less than $0.10 per article per year to provide access to all the open source scholarly articles on the internet
- A scholar will typically pay $0.50 per print article per year to have a journal subscription containing the articles of interest
- A scholar will typically pay less than $0.05 to access and print an open source article from the internet.

These are examples of “transaction costs”, the cost of exchanging or transferring information from one point to another. The internet only / open source model lowers transaction costs across the board for stakeholders by factors of between 5 and 20. The total cost to publish an article online in the JACMP is approximately $300 per article. Presently, that cost is borne by our advertisers, and that is how we provide the JACMP to a worldwide audience, to anyone with web access.

Our advertisers also experience the benefit of lowered transaction costs. Generally, it costs less to buy a pair of eyes to look at a vendor web site in the electronic media than in the print media. I sincerely hope you will support our vendors and use the vendor links in the JACMP. We track the number of click‐throughs in order to justify to our vendors the cost of a banner ad. If you just make it a habit to visit vendor web sites using JACMP banner ads, it helps us keep the journal available to you without cost.

Let me express my very best wishes and thanks to Walter Huda and John Horton for their dedicated support as Associate Editors over the past several years. Both are leaving the JACMP, and we appreciate all the volunteer work they have done.

Michael D. Mills, PhD

### The Journal of Applied Clinical Medical Physics

**Table:** 

**Editorial Board 2005**	
Editor‐in‐Chief	Michael D. Mills	
Deputy Editor‐in‐Chief	Timothy D. Solberg	
Associate Editor‐at‐Large	Richard Stark	
**Associate Editors**	
Radiation Oncology Physics	Nzhde Agazaryan, B. Gino Fallone
	John Gibbons, Michael Herman
	Edwin McCullough, Janelle Molloy
	Matthew Podgorsak, Nikos Papanikolaou
	Mehrdad Sarfaraz, Lu Wang
Medical Imaging	Walter Huda, William Pavlicek
Radiation Measurements	Larry DeWerd
Radiation Protection & Regulations	James Deye
Nonionizing Topics	Bhudatt Paliwal
Other Topics	Timothy Solberg
Book/Media Reviews	James Smathers
Editorials	John Horton
**Journal Production Team**	
Proofreader	Heather Shand
Copy Editor	Betty R. Robinson
Layout Editor	Laura Shand
Manuscript Manager	Patricia Walker

